# Compound Cement and Amalgam Filling

**Published:** 1897-06

**Authors:** H. Baldwin

**Affiliations:** Eng.


ARTICLE VI.

### COMPOUND CEMENT AND AMALGAM FILLING.

BY DR. H. BALDWIN, ENG.

The *modus operandi* is as follows: The cavity should be excavated with the usual care as regards removal of the decay, but the amount of undercutting which is necessary is very much less than for either amalgam or gold. The cavity should be thoroughly dried. The amalgam should first be mixed and of a convenient sort of consistency. The cement should then be mixed and of a decidedly thin consistency, not much thicker than would be used for fixing crowns. The cavity should then be filled with the cement, preferably by means of the same spatula as has been used for mixing it. Then immediately a large piece of the amalgam should be pressed into the cement, and, by means of a smooth, rounded instrument, should be driven home, working from the center to the circumference, and so expressing much of the cement on all sides. The edges of the cavity should then be quickly cleared of both cement and amalgam, by means of spoon or other excavators, till not a trace of anything is left at any of the edges, especially at the cervical edge, if the cavity is an interstitial one. This obviates the danger of getting the cement exposed on the surface when the work is finished. The remaining cavity should then be filled up with pure amalgam carrying it down to the cervical edge in small pieces with perhaps a trifle more mercury added so as to ensure its going down completely, and then finishing with harder amalgam and squeezing with bibulous paper in the well-known way. A matrix should be used in large composite cavities and may be applied either before commencing to fill or immediately after packing the first pieces of amalgam and clearing the edges. Putting on the matrix after clearing the edges keeps the matrix clean and free from cement.

Cavities for which this composite filling is suitable are practically all those which are generally considered suitable for cement alone; many cases which are generally considered suitable for cement alone, and in addition many which would otherwise be suitable only for gold. All large interstitial cavities in molars and bicuspids and crown cavities which are fit to receive a hard filling at all may with propriety be filled by this method. There is little in common between a filling of this sort and an ordinary amalgam filling. Ordinary amalgam as a filling material is open to many objections which the combination is entirely free from, and the combination presents a number of merits which belong to it alone. Thus, to compare it, point by point, with gold or amalgam: (1) it requires a much smaller sacrifice of healthy tooth substance; (2) it leaves a stronger tooth; (3) it necessitates much less pain in excavating; (4) valuable time is saved in excavating; (5) it interposes a non-conducting layer between the sensitive dentine and the metal; (6) it adheres to the cavity; (7) it is more water-tight; (8) compared with amalgam, at all events, it does not stain the tooth, nor show through the thin enamel of a nasty color, and (9) it is quickly done.

I venture to submit: (1) that all cement fillings in back teeth which we so often meet with as permanencies would be better treated by coating the cement with amalgam in this way; (2) that most teeth which are filled with cement as a trial for a temporary purpose would be better filled as a permanency in this way. Where the cement will be tolerated this combination will equally be tolerated, and whereas it is exceedingly difficult often to pack simple cement tightly against the cervical portion of a deep interstitial cavity it is easy, by means of the amalgam, to drive the cement well home. In passing, I would like to give it as my opinion that the supposed tendency of cement to undergo especially rapid solution at the cervical edge does not exist. The disappearance of the cement and appearance of a cavity in this situation is due to the cement never having been in absolute apposition with the tooth at the point, or to the decay there never having been thoroughly removed. The difficulty of packing plain cement at this point is not, I fancy, generally realized, and lies not only in the remoteness of the situation, but in the fact that a little moisture frequently bedews that part, and that the gum presents a prominent and possibly overhanging edge, which edge, when pressed on, is especially liable to give forth a serous or sanious oozing; (3) that nearly every amalgam filling would be improved by being inserted in this way; I have used this method with gradually increasing frequency since my early days of practice, thirteen years ago, and to-day I hardly ever put in an amalgam without the preliminary adhesive stratum of cement. Of course, care and neatness are necessary in this as in every dental operation, and it does not do to leave a layer of cement outcropping at the edges.—*British Dental Journal.*

